# Association between sedentary behavior and depression among Japanese medical students during the COVID-19 pandemic: a cross-sectional online survey

**DOI:** 10.1186/s12888-022-03997-x

**Published:** 2022-05-20

**Authors:** Tsubasa Tashiro, Noriaki Maeda, Shogo Tsutsumi, Makoto Komiya, Satoshi Arima, Rami Mizuta, Kazuki Fukui, Yuichi Nishikawa, Yukio Urabe

**Affiliations:** 1 Graduate School of Biomedical and Health Sciences Division of Integrated Health Sciences, Hiroshima University, Hiroshima, 734-8553 Japan; 2 Faculty of Frontier Engineering, Institute of Science & Engineering, Kanazawa University, Kanazawa, 920-1192 Japan

**Keywords:** COVID-19, Japan, Medical students, Sedentary behavior, Depression, Screen time, Online survey, Pandemic

## Abstract

**Background:**

Sedentary behavior, particularly prevalent during the 2019 coronavirus disease (COVID-19), has been shown to lead to depression. In medical students, this could negatively affect the provision of healthcare. Thus, this study aimed to investigate the association between sedentary behavior and depression in Japanese medical students during the COVID-19 pandemic.

**Methods:**

An online cross-sectional survey was conducted from July 30 to August 30, 2021, using anonymous questionnaire system to assess respondents’ sociodemographic characteristics, physical activity, sedentary behavior among 1000 Japanese university students. Depression was assessed using the Patient Health Questionnaire-2 (PHQ-2). The data of 484 respondents (48.4%) were included in a stepwise analysis, where we set the difference between medical and non-medical students as Model 1 and that between medical students with and without depression as Model 2. For group comparisons of both models, the chi-square test was used for sociodemographic characteristics, and the Mann–Whitney U-test was used for physical activity and sedentary behavior. In Model 2, factors associated with depression among medical students were analyzed by logistic regression analysis.

**Results:**

In Model 1, medical students were less physically active (*p* < 0.001), had longer sedentary time (*p* < 0.001), and had higher PHQ-2 scores (*p* = 0.048) than non-medical students. In Model 2, medical students with depression had longer sedentary time (*p* = 0.004) and longer leisure screen time than those without depression (*p* = 0.007). Moreover, logistic regression analysis adjusted for potential confounders showed that sedentary time (OR = 1.001, *p* = 0.048) and leisure screen time (OR = 1.003, *p* = 0.003) were significantly associated with depression among medical students.

**Conclusions:**

Based on these results, it is evident that reducing Japanese medical students’ sedentary time and leisure screen time can help combat depression during the COVID-19 pandemic; thus, these results can guide the development of appropriate interventions to prevent and treat depression.

## Background

The coronavirus disease 2019 (COVID-19) pandemic has become a global public health emergency. The World Health Organization (WHO) advised the public to avoid crowds and close contact with others to prevent the transmission of COVID-19 [1]. In the field of education, classes were switched to online courses and students were forced to stay and study at home, which resulted in their isolation from society. Although self-isolation prevents infection [2], these strategies may lead to an increase in depressive symptoms as a synergistic effect with the fear of COVID-19 [3]. Indeed, a recent meta-analysis suggests that mental health problems among students seem to have worsened in the wake of the COVID-19 pandemic [4]. Thus, comprehensive approaches to reduce mental health problems for students are required during the COVID-19 pandemic [5].

Sedentary behavior is related to depressive symptoms [6], while physical activity is related to a lower incidence and prevalence of depression [7]. After the onset of the COVID-19 pandemic, an epidemiological study on 3052 US adults reported increased sedentary behavior accompanied by decreased physical activity [8]. Another study among 500 000 Spanish university students showed that screen time (leisure, study, and work) increased, in addition to increased sedentary time and decreased physical activity [9]. Prolonged screen time-based sedentary behavior can affect the prevalence of depression by impairing biological functions such as central nervous system arousal and sleep disorders [10]. These factors could have accelerated the onset of depression, as in the case of COVID-19, which spread rapidly worldwide.

According to a systematic review of 195 studies among 129 123 medical students from 47 countries, 27.2% screened positive for depression [11]. This rate was higher than the prevalence of depression (19.0% in males and 22.0% in females) reported in a comparative study on 17 348 university students from 23 countries [12]. A recent meta-analysis of health professionals reported a 31.8% prevalence of depression, which may be a future mental health issue for medical students [13]. Prospective studies have suggested that depressive symptoms in medical students could have negative effects on the quality of healthcare services provided by those students in medical institutions [14, 15]. Moreover, considering the impact of COVID-19 threats on sedentary behavior and depression, it is vital to understand these relationships in medical students.

However, to the best of our knowledge, few studies have investigated the relationship between sedentary behavior and mental health in medical students during the COVID-19 pandemic. Hence, the purpose of this study was to address this gap by investigating this relationship among Japanese medical students.

## Methods

### Study design and setting

**Fig. 1:**
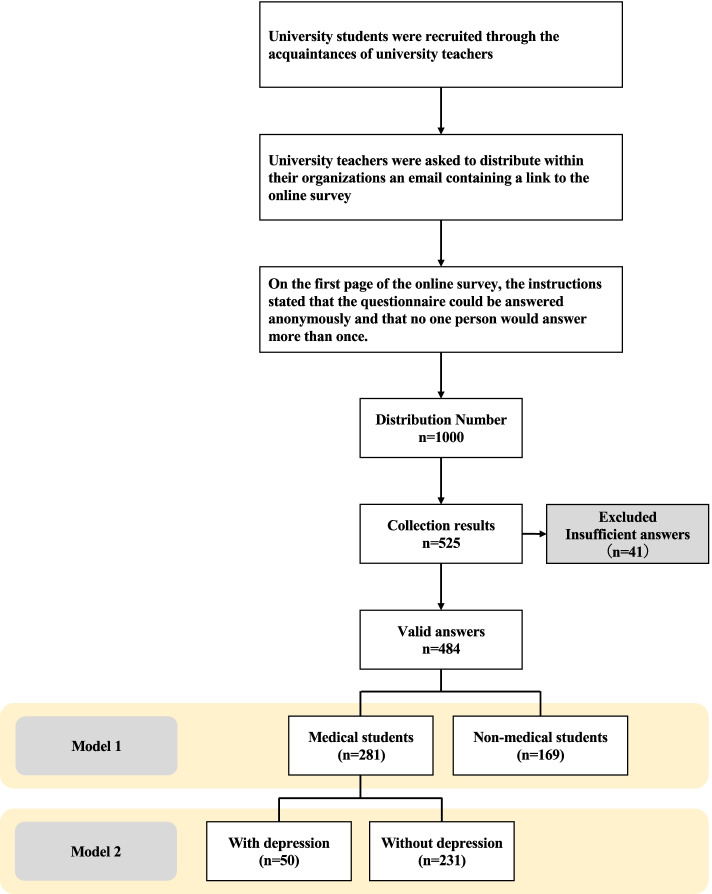
Flowchart of the process of participant recruitment and data analysis

This was an observational, cross-sectional study. An online survey was conducted from July 30 to August 30, 2021, using Google Forms (Alphabet, Mountain View, CA, USA). The target population was Japanese university students who belonged to universities in the Chubu, Kanto, Kinki, Shikoku, Chugoku, and Kyushu regions. The university students were recruited through the acquaintances of university teachers. That is, we asked the university teachers to distribute an email within their organization with a Google Forms link to the online survey. On the first page of the online survey, the instructions stated that the questionnaire could be answered anonymously and that no one person could answer more than once. On the same page, the study was explained and only those who agreed to complete the online survey could proceed to the next page. We structured the multiple-choice questions to collect accurate data, which was the minimum number of questions. The inclusion criteria were individuals who (a) were at least 18 years old, (b) were enrolled in a university, (c) resided in Japan at the time of the pandemic, and (d) agreed to provide informed consent. Informed consent for this survey was obtained through the agreement text and checkboxes on the first screen of the Google form. The exclusion criterion was respondents who took psychiatric medication daily. The final sample comprised 1000 Japanese university students. Figure 1 is a flowchart showing the recruiting process of the participants for this study.

This study was based on the recommendations of the Checklist for Reporting Results of Internet E-Surveys (CHERRIES) [16]. Furthermore, it conformed to the guidelines of the Declaration of Helsinki and all its future amendments and was approved by the Epidemiology Ethics Committee of Hiroshima University (Approval ID: E-2531).

### Instruments and data collection

The questionnaire included sociodemographic data such as age, gender (male/female), body mass index (BMI) calculated using height and weight (kg/m^2^, normal/underweight/overweight or obese), living status (alone/with others), marital status (married/not married), pet status (Yes/No), part-time job status (Yes/No), confinement status caused by COVID-19 (strict/except purchase or work/no) [17], and faculty (medical/non-medical).

Physical activity and sedentary time were assessed using the International Physical Activity Questionnaire – Short Form (IPAQ-SF) [18]. We assessed vigorous physical activity, moderate physical activity, and walking time on average in a week; the total physical activity was subsequently calculated (Mets*mins/week). Respondents were also questioned about their sedentary time on weekdays. In addition, we categorized screen time (min/day) as two conditions: leisure and study [19].

The questionnaire included a screening test for psychosocial disturbances related to depressive symptoms in the past two weeks. The Patient Health Questionnaire 2 (PHQ-2) was used to assess the frequency of depressed mood and anhedonia [20, 21]. Responses were rated on a 4-point Likert scale, which ranged from 0 (not at all) to 3 (nearly every day), with a maximum score of 6. A score of ≥ 3 was defined as screening positive for depressive symptoms.

### Statistical analysis

The analysis was performed using a phased process with two models (Fig. 1). The respondents were divided into two groups in Model 1: medical students and non-medical students. Subsequently, sociodemographic characteristics, physical activity, sedentary time, screen time, and PHQ-2 were compared between these two groups. Model 2 differentiated between medical students with and without depression according to their PHQ-2 scores. For the data analysis of Model 2, we compared sociodemographic characteristics, physical activity, sedentary time, and screen time between these two groups. The Shapiro–Wilk test was performed before each analysis to confirm normality. Moreover, the chi-square test was used for sociodemographic characteristics, and the Mann–Whitney U-test was used to compare physical activity, sedentary time, and screen time for both models. In the chi-square test, a Bonferroni correction was performed to test multiple categories. A logistic regression analysis was conducted to determine the factors that influenced depression among medical students; a PHQ-2 score of ≥ 3 was coded as 0 and ≤ 2 as 1. The dependent variable was the presence or absence of depression, and the independent variables were sedentary time and leisure screen time (crude model). Additionally, it was adjusted by gender and living status (adjusted model). Odds ratios (OR) and 95% confidence intervals (CIs) were calculated for the dependent variables. The variance inflation coefficient was calculated to evaluate the possibility of multicollinearity of the independent variables in the multivariate regression analysis.

A previous study recommended that the number of participants per variable should be ≥ 10 [22]. The sample size for the logistic regression analysis was predetermined using three independent parameters and suggested that this study required 10 times as many participants. Therefore, at least 30 participants were required in each group (medical and non-medical students). All data were analyzed using IBM SPSS Statistics for Windows (version 23.0; IBM Corp., Armonk, NY, USA). The significance level was set at *p* < 0.05.

## Results

### Sociodemographic characteristics among medical and non-medical students

Of the 1000 respondents, 525 (52.5%) responded and 41 were excluded due to incomplete answers or obvious errors. Ultimately, 484 respondents (48.4%) were included. In Model 1, the mean ages of the medical and non-medical students were 21.7 years (SD = 3.4) and 20.8 years (SD = 2.2), respectively. Of the respondents, 39.0% were male and 61.0% female. Regarding BMI, 78.9% were normal weight, 10.7% were overweight or obese, and 10.3% were underweight, with a significant difference between medical and non-medical students (*p* < 0.001). There were no significant differences between the groups regarding whether they lived alone (50.8%), were married (1.0%), had a pet (15.1%), or worked part-time (53.9%). In this sample, 10.1% were strictly restricted from going out, 78.9% were restricted except for purchases and work, and 11.0% lived without restrictions during the COVID-19 pandemic (Table 1).

**Table 1:** Sociodemographic characteristics of medical and non-medical students

	Model 1					
	*N* (%)	Medical students (*n* = 281)	Non-medical students (*n* = 203)	χ^2^	*p* value	Cramer’s V
Gender						
Male	189 (39.0)	103	86	1.614	0.204	0.06
Female	295 (61.0)	178	117			
BMI						
Normal	382 (78.9)	232*	150	15.587	< 0.001	0.18
Underweight	50 (10.3)	32	18			
Overweight or obese	52 (10.7)	17*	35			
Living status						
Alone	246 (50.8)	146	100	0.343	0.558	0.03
With others	238 (49.2)	135	103			
Marital status						
Married	5 (1.0)	5	0	3.650	0.056	0.09
Not married	479 (99.0)	276	203			
Pet						
Yes	73 (15.1)	44	29	0.173	0.677	0.02
No	411 (84.9)	237	174			
Part-time job						
Yes	261 (53.9)	210	151	0.008	0.931	0.00
No	123 (25.4)	71	52			
Confinement						
Strict	49 (10.1)	30	19	5.296	0.071	0.11
Except purchase or work	382 (78.9)	228	154			
No	53 (11.0)	23*	30			

*BMI* Body mass index. *Chi-square test (*p* < 0.05) with Bonferroni correction for multiple categories

### Physical activity, screen time, and depressive symptoms reported by medical and non-medical students

The total physical activity, vigorous physical activity, and moderate physical activity of medical students were significantly lower than those of non-medical students (*p* < 0.001, *p* < 0.001, and *p* = 0.006, respectively). No significant differences were observed between the groups regarding walking (*p* = 0.809). Moreover, sedentary time was longer in medical than non-medical students during the COVID-19 pandemic (*p* < 0.001). Screen time spent on leisure did not differ between the groups, whereas screen time spent on study was longer in medical students (*p* = 0.035). Regarding depressive symptoms, the PHQ-2 value in medical students was higher than that in non-medical students (*p* = 0.048) (Table 2).

**Table 2:** Physical activity, screen time, and depressive symptoms reported by medical and non-medical students

Variables	Model 1			
	Medical students	Non-medical students	*p* value	*r*
IPAQ-SF				
Total PA (Mets*mins/week)	1220.0 [497.5–2568.0]	2034.0 [855.0–5500.0]	< 0.001	0.22
Vigorous PA (Mets*mins/week)	120 [0.0–960.0]	960.0 [0.0–3600.0]	< 0.001	0.27
Moderate PA (Mets*mins/week)	0.0 [0.0–480.0]	120.0 [0.0–640.0]	0.006	0.13
Walking (Mets*mins/week)	396.0 [150.0–792.0]	396.0 [132.0–900.0]	0.809	-0.01
Sedentary Time (min/day)	360.0 [240.0–540.0]	300.0 [180.0–480.0]	< 0.001	-0.19
Screen Time				
Leisure (min/day)	200.0 [120.0–300.0]	200.0 [120.0–360.0]	0.569	0.03
Study (min/day)	90.0 [60.0–150.0]	60.0 [30.0–120.0]	0.035	-0.10
Depressive Symptoms				
PHQ-2	1.48 ± 1.46	1.14 ± 1.11	0.048	-0.09

Data are expressed as medians [interquartile range]. *r* means effect size, *IPAQ-SF* International Physical Activity Questionnaire, *PHQ-2* Patient Health Questionnaire

### Sociodemographic characteristics among medical students with and without depression

The mean ages of the groups with and without depression were 21.6 years (SD = 3.5) and 21.9 years (SD = 3.4), respectively, in Model 2. The sample was 36.7% male and 63.3% female. Normal BMI was identified in 82.6%, underweight in 11.4%, and overweight or obese in 6.0% of students. Approximately half of the respondents (52.0%) answered that they lived alone. Among the respondents, 15.7% had pets and 74.7% worked part-time. The highest percentage of confinement due to the COVID-19 pandemic was except for purchase and work (81.1%), followed by strict (10.7%) and no restrictions (8.2%). No significant differences were found for any of the items (Table 3).

**Table 3:** Sociodemographic characteristics of medical students with and without depression

	Model 2					
	*N* (%)	With depression (*n* = 50)	Without depression (*n* = 231)	χ^2^	*p* value	Cramer’s V
Gender						
Male	103 (36.7)	12	91	4.195	0.041	0.12
Female	178 (63.3)	38	140			
BMI						
Normal	232 (82.6)	41	191	0.023	0.989	0.01
Underweight	32 (11.4)	6	26			
Overweight or obese	17 (6.0)	3	14			
Living status						
Alone	146 (52.0)	27	119	0.102	0.750	0.02
With others	135 (48.0)	23	112			
Marital status						
Married	5 (1.8)	0	5	1.102	0.294	0.06
Not married	276 (98.2)	50	226			
Pet						
Yes	44 (15.7)	5	39	1.475	0.225	0.07
No	237 (84.3)	45	192			
Part-time job						
Yes	210 (74.7)	38	172	0.052	0.820	0.01
No	71 (25.3)	12	59			
Confinement						
Strict	30 (10.7)	7	23	0.706	0.703	0.05
Except purchase or work	228 (81.1)	39	189			
No	23 (8.2)	4	19			

*BMI* Body mass index

### Physical activity and screen time reported by medical students with and without depression

There were no significant differences between the groups with and without depression in total physical activity, vigorous physical activity, moderate physical activity, and walking. Sedentary behavior was significantly longer in the group with depression than in the group without depression (*p* = 0.004). Moreover, screen time spent on leisure was significantly longer in the group with depression (*p* = 0.007), whereas no significant difference was found among the groups regarding screen time spent on study and work (Table 4).

**Table 4:** Physical activity and screen time reported by medical students with and without depression

Variables	Model 2				
	With depression		Without depression	*p* value	*r*
IPAQ-SF					
Total PA (Mets*mins/week)	946.0 [290.0–2186.3]	1333.0 [594.0–2598.0]		0.067	-0.11
Vigorous PA (Mets*mins/week)	0.0 [0.0–840.0]	240.0 [0.0–960.0]		0.270	-0.07
Moderate PA (Mets*mins/week)	0.0 [0.0–340.0]	0.0 [0.0–480.0]		0.341	-0.06
Walking (Mets*mins/week)	340.0 [99.8–615.0]	450.0 [187.5–875.0]		0.160	-0.08
Sedentary Time (min/day)	480.0 [300.0–615.0]	360.0 [180.0–500.0]		0.004	0.17
Screen Time					
Leisure (min/day)	270.0 [150.0–435.0]	180.0 [120.0–300.0]		0.007	0.16
Study (min/day)	65.0 [58.3–180.0]	90.0 [60.0–130.0]		0.862	0.01

Data are expressed as medians [interquartile range]. *r* means effect size, *IPAQ-SF* International Physical Activity Questionnaire

### Multiple logistic analysis for Model 2

To assess the determinants associated with depressive symptoms, a multiple logistic analysis was conducted using the PHQ-2 score as an observational variable. Statistically significant associations were detected for sedentary time (β = 0.001, *p* = 0.036, OR = 1.001, 95% CI 1.000–1.003) and screen time for leisure (β = 0.001, *p* = 0.002, OR = 1.003, 95% CI 1.001–1.005) in crude model. In the adjusted model with gender and living status as potential confounders, significant associations were detected for sedentary time (β = 0.001, *p* = 0.048, OR = 1.001, 95% CI 1.000–1.003) and screen time for leisure (β = 0.003, *p* = 0.003, OR = 1.003, 95% CI 1.001–1.005) as well (Table 5).

**Table 5:** Multiple logistic analysis for Model 2

Variables	β	SE	Wald	df	*p* value	Odds ratio	95% CI	
							Lower	Upper
*Crude model*								
Sedentary time	0.001	0.001	4.414	1	0.036	1.001	1.000	1.003
Screen time (Leisure)	0.001	0.001	9.408	1	0.002	1.003	1.001	1.005
*Adjusted model*								
Sedentary time	0.001	0.001	3.913	1	0.048	1.001	1.000	1.003
Screen time (Leisure)	0.003	0.001	9.006	1	0.003	1.003	1.001	1.005
Gender	0.689	0.372	3.428	1	0.064	1.991	0.960	4.127
Living status	-0.117	0.331	0.124	1	0.725	0.890	0.465	1.703

*SCI* confidence interval, Variance inflation factor: Sedentary time: 1.036; Screen time (Leisure): 1.037; Gender: 1.025; Living status: 1.024

## Discussion

To the best of our knowledge, this is the first study to explore the relationship between sedentary behavior and depressive symptoms in Japanese medical students during the COVID-19 pandemic. The cumulative number of COVID-19 cases in Japan was 1 708.742 as of October 11, 2021 [23], with cases still increasing more than a year after the first infection. Our findings in such a situation emphasized that the COVID-19 pandemic heightened the depressive symptoms of Japanese medical students. That is, they were less physically active and more depressed than non-medical students. Additionally, we found that medical students with depression had more sedentary lifestyles than those without depression. Lengthy sedentary time and leisure screen time may be part of early depression.

According to the analysis of Model 1, medical students were less physically active than non-medical students. Medical students are under a great deal of daily mental stress due to pressure to maintain excellent academic performance [24]. Moreover, medical students have little time for regular physical activity due to their demanding studies [25]. Thus, this was a reasonable finding. Overlapping with this, the forced confinement due to the COVID-19 pandemic may have led them to living a more sedentary lifestyle. Similar to previous studies that showed that the COVID-19 pandemic was linked to sedentary behavior and psychological distress in university students [3, 26], our results suggested that medical students with longer sedentary time had worse depressive symptoms. Other recent research showed that students’ mental health, including depression, during the pandemic was worse than during the pre-pandemic and that they globally suffered a disproportionate burden of psychological health issues [4].

The analysis of Model 2, which focused on medical students, revealed that depressive symptoms were more prevalent among females than males. In general, it is known that a gender difference exists in the prevalence of depression [27]. The fact that gender was not detected as a determinant of depression in our multiple logistic analysis supports previous studies on medical staff during the COVID-19 outbreak [28]. Thus, gender may not have been a deciding factor in the development of depression among Japanese medical students during the COVID-19 pandemic. Additionally, longer sedentary behavior and screen time for leisure impacted depressive symptoms in medical students. Our cross-sectional study suggests that the length of those times could be part of early depression. In contrast, no statistical difference was found for physical activity, which included total physical activity, vigorous activity, moderate physical activity, and walking, between medical students with and without depression.

A meta-analysis conducted early in the COVID-19 pandemic suggests that mental health problems among health professionals were exacerbated and that more attention should be paid to psychosocial impacts [13]. The causal association between confinement due to the COVID-19 pandemic and depressive symptoms could not be examined in this study. However, the results indicated that sedentary behavior exacerbated depressive symptoms as a result of decreased physical activity due to restrictions on going out. In medical students with depression, the sedentary behavior and screen times were 480.0 and 270.0 min per day, respectively. These figures were comparatively higher than those of a recent systematic review that involved 125 studies on university students globally (437.4 min per day of sedentary time, 135.6 min per day of screen time on smartphones, and 122.4 min per day of screen time on computers and video games) [29]. In a 2014 meta-analysis of the general population, the relative risk of depression was found to be 1.31 (95% CI 1.16–1.48) for sedentary behavior. Additionally, long-term TV viewing and computer or Internet use were cited as factors that increased this risk [30]. A previous meta-analysis also suggested that Internet addiction was significantly associated with depression (OR = 2.77) [31]. This suggests that Japanese medical students should reconsider their behavior at home during confinement to mitigate depression. Moreover, a recent study indicated that prolonged sedentary time was more important than total sedentary time [32]. Therefore, there may be a need to manage recreational screen time among medical students.

In Japan, online classes were launched at universities with the issuance of the first emergency declaration in 2020. Since then, online learning has become more common among university students. In ordinary situations, increasing opportunities for outdoor social interaction may help reduce depressive symptoms. However, during an emergency such as the current pandemic, improving mental problems through online interventions at home should be a priority. In recent years, web-based cognitive behavioral therapy has been highlighted as a way to improve psychiatric symptoms among university students [33]. Notably, university students use the Internet daily [34]. Therefore, future studies on web-based cognitive behavioral therapy interventions and their impact on depression are expected to provide medical students with health promotion and prevention strategies that they can easily use at home.

This study has some limitations. First, the presence or absence of depression was not determined based on a clinical examination. However, the PHQ-2 has been proven to be valid tool for depression screening [20]. Second, as all the items were self-reported, recall bias and the respondent’s social setting may have affected the results. Third, as this study had a cross-sectional research design, we were unable to conduct a longitudinal study on the occurrence of depression and sedentary lifestyles. Additional research is necessary to investigate the long-term effects of the sedentary lifestyles of Japanese medical students on depressive symptoms in a large population.

## Conclusions

Our study investigated the association between sedentary behavior and depression among Japanese medical students during the COVID-19 pandemic. Medical students had lower physical activity and higher PHQ-2 scores for depression screening compared to non-medical students. A secondary analysis showed that, among medical students, the prevalence of depression was higher in females than in males. Additionally, medical students with depression had more sedentary behaviors and screen time for leisure compared to medical students without depression. A multiple logistic analysis revealed that longer sedentary time and screen time for leisure were significantly associated with depression among medical students. Long-term sedentary behavior could be part of early depression, and this study indicates a potential need to prevent future mental health problems in healthcare professionals. Taken together, these findings strongly suggest the need to control the increase in sedentary time and screen time for leisure among Japanese medical students during the COVID-19 pandemic.

## Data Availability

The data used and analyzed in this study are available from the corresponding author upon reasonable request.

## Abbreviations

BMI: Body mass index
CI: Confidence interval
COVID-19: Coronavirus disease 2019
IPAQ-SF: International Physical Activity Questionnaire – Short Form
PHQ-2: Patient Health Questionnaire-2
OR: Odds ratio
